# Supplementary material for: Splice-Junction-Based Mapping of Alternative Isoforms in the Human Proteome
Source: Cell Rep. Author manuscript; Available in PMC 2020 Jan 15. (PMC6961840; doi:10.1016/j.celrep.2019.11.026)
Supplement: 2 [file NIHMS1546469-supplement-2.zip › DF1/PXD006675/LeftAtrium/LeftAtrium_51_TTN_APHVEFLR.pdf]

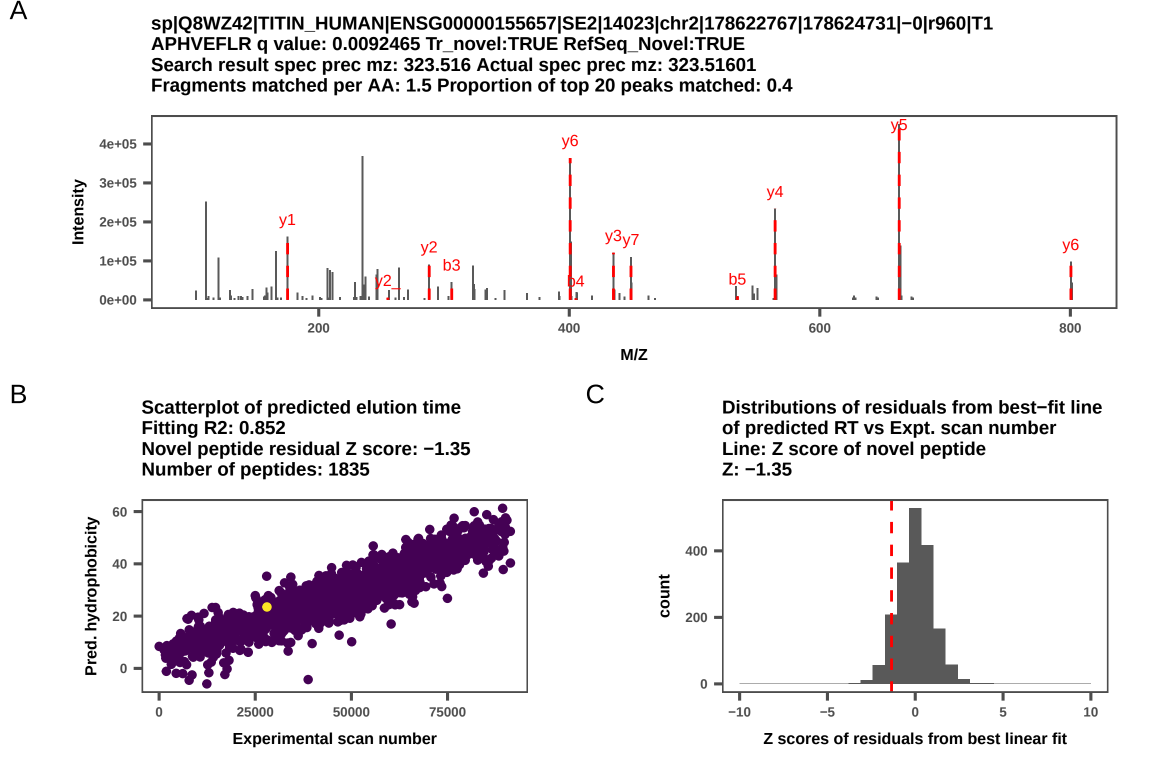
A
sp|Q8WZ42|TITIN_HUMAN|ENSG00000155657|SE2|14023|chr2|178622767|178624731|−0|r960|T1
APHVEFLR q value: 0.0092465 Tr_novel:TRUE RefSeq_Novel:TRUE
Search result spec prec mz: 323.516 Actual spec prec mz: 323.51601
Fragments matched per AA: 1.5 Proportion of top 20 peaks matched: 0.4
Intensity
4e+05
3e+05
2e+05
1e+05
0e+00
y1
y2_
y2
b3
y6
b4
y3
y7
b5
y4
y5
y6
200
400
600
800
M/Z
B
Scatterplot of predicted elution time
Fitting R2: 0.852
Novel peptide residual Z score: −1.35
Number of peptides: 1835
Pred. hydrophobicity
60
40
20
0
0
25000
50000
75000
Experimental scan number
C
Distributions of residuals from best−fit line
of predicted RT vs Expt. scan number
Line: Z score of novel peptide
Z: −1.35
count
400
200
0
−10
−5
0
5
10
Z scores of residuals from best linear fit
